# Efficacy and Safety of Photobiomodulation Therapy for Motor Symptoms in Parkinson’s Disease: A Systematic Review and Meta-Analysis

**DOI:** 10.3390/brainsci16090925

**Published:** 2026-08-31

**Authors:** Joao Mariano, Eneidy Piña Mojica, Andrés Soto-Rodríguez, Anna Carolyna Gianlorenço, Kevin Pacheco-Barrios, Felipe Fregni

**Affiliations:** 1 Neuromodulation Center and Center for Clinical Research Learning, Harvard Medical School, Spaulding Rehabilitation Hospital, Boston, MA 02129, USAeneidy.pina31@gmail.com (E.P.M.); asoto@rmihealth.com (A.S.-R.);; 2 Research Unit, The Regenerative Medicine Institute, Avenida Escazú, Torre Lexus 206, San José 10203, Costa Rica; 3 Neuroscience and Neurological Rehabilitation Laboratory, Physical Therapy Department, Federal University of São Carlos, São Carlos 13565-905, Brazil; 4 Vicerrectorado de Investigación, Unidad de Investigación para la Generación y Síntesis de Evidencias en Salud, Universidad San Ignacio de Loyola, Lima 15023, Peru

**Keywords:** photobiomodulation, Parkinson’s disease, motor function, systematic review

## Abstract

**Highlights:**

**What are the main findings?**

- Meta-analyses of MDS-UPDRS-III (k = 4), TUG (k = 3), and walking speed (k = 2) found no statistically significant pooled effects, with no outcome exceeding its respective MCID threshold.
- Other motor outcomes, including UPDRS, presented no significant improvements.

**What are the implications of the main findings?**

- PBM was safe and well tolerated in Parkinson’s disease, with no serious adverse events reported.
- High adherence rates (>90% across all reporting trials) confirmed the feasibility and patient acceptance of PBM.
- Future trials should employ standardized reporting, incorporate biomarkers of target engagement, and be adequately powered against established MCID thresholds.

**Abstract:**

**Background:** Parkinson’s disease (PD) is a progressive neurodegenerative disorder causing disabling motor dysfunction and substantial socioeconomic burden. Photobiomodulation (PBM), a noninvasive red/near-infrared light therapy, has shown preclinical promise, but its clinical efficacy and safety in PD remain uncertain. **Objectives:** To systematically evaluate the efficacy and safety of PBM on motor outcomes in PD. **Methods:** This PRISMA-compliant systematic review was prospectively registered in PROSPERO (CRD420261411269). Databases were searched through May 2026. Randomized and non-randomized trials in adults with PD were included. Two reviewers independently screened studies, extracted data, and assessed risk of bias (RoB 2.0; ROBINS-I). Where data permitted, random-effects meta-analyses used mean difference (MD) with REML estimation and Hartung–Knapp–Sidik–Jonkman confidence intervals. **Results:** Eight trials (n = 279) were included, exhibiting substantial heterogeneity in wavelength, device type, stimulation sites, and dosimetry. PBM was safe and well tolerated, with only minor adverse effects. Meta-analysis of four RCTs found no significant effect on MDS-UPDRS-III (MD = −1.37 points, 95% CI: −5.55 to 2.80; I^2^ = 20.6%) or TUG (MD = −0.52 s, 95% CI: −5.03 to 3.99; I^2^ = 55.1%). An exploratory TMWT meta-analysis (k = 2) yielded MD = −0.65 s (95% CI: −1.78 to 0.49; I^2^ = 0.0%), with direction consistently favoring PBM. Individual effect sizes ranged from negligible (d = 0.03–0.06) to small-to-moderate (d = 0.41). **Conclusions:** PBM appears safe, feasible, and acceptable in PD, but current evidence does not support clinically meaningful motor benefits. Heterogeneous protocols, small samples, and absent target-engagement biomarkers limit definitive interpretation. Well-powered standardized trials with mechanistic endpoints are needed.

## 1. Introduction

Parkinson’s disease (PD) is a progressive neurodegenerative disorder characterized by both motor and non-motor manifestations resulting from degeneration of dopaminergic neurons in the substantia nigra pars compacta and disruption of basal ganglia circuits. As dopamine levels decline, movement becomes increasingly difficult to initiate, scale, and sustain, generating the motor symptoms for which the disease is best known [1,2]. Bradykinesia, rigidity, tremor, and postural instability are its four major features. However, non-motor symptoms—including autonomic dysfunction, mood disturbances, cognitive decline, sleep abnormalities, and sensory alterations—frequently precede motor onset and substantially affect quality of life. These manifestations are also indicative of widespread extranigral pathology and frequently become the predominant cause of disability as the disease progresses [2,3].

PD is the second most prevalent neurodegenerative disorder worldwide, affecting approximately 11.8 million individuals, with nearly 1 million cases in the United States [4]. Furthermore, annual expenditures related to PD amount to an estimated $51.9 billion, with $26.5 billion allocated to rehabilitation and clinical care [5]. Its prevalence rises in parallel with population aging (typically over 60 years of age), imposing a growing socioeconomic burden. Beyond direct healthcare expenditures, the chronic and progressive nature of PD requires long-term medical care, rehabilitation strategies, and caregiver support [6,7,8]. As disease-modifying therapies remain unavailable, management focuses on symptomatic control. Levodopa and dopamine agonists effectively improve motor symptoms but are limited by long-term complications such as motor fluctuations and dyskinesias [9,10]. Non-pharmacological interventions, including physical therapy and deep brain stimulation, may enhance functional outcomes but do not alter disease progression and are not suitable for all patients. These limitations have motivated continued exploration of therapeutic strategies targeting cellular circuit-level mechanisms implicated in PD pathophysiology [11,12,13,14].

Photobiomodulation (PBM) is a noninvasive therapeutic approach involving the application of red or near-infrared light, typically within wavelengths of 600–1000 nm. Photons are absorbed by cytochrome C oxidase within the mitochondrial electron transport chain, potentially increasing adenosine triphosphate production, modulating oxidative stress, influencing intracellular calcium signaling, and attenuating inflammatory processes [15,16,17]. Through these light-mediated biological mechanisms, PBM has been hypothesized to support neuronal energy metabolism and cellular resilience; however, the extent to which these biological effects translate into meaningful clinical benefits remains uncertain [18,19,20]. Translational studies in MPTP-induced non-human primate models have also reported attenuation of dopaminergic neuronal loss and motor impairment following near-infrared light exposure, providing an anatomical and physiological approximation to human disease [21].

Translational efforts following rodent and non-human primate studies have led to exploratory applications of PBM in humans [22,23]. Early reports, although limited by small sample sizes and uncontrolled designs, suggested that sustained and repeated daily exposure may be necessary to observe measurable clinical changes. Despite its biological rationale, the clinical efficacy remains to be established.

Safety and tolerability are additional considerations in PBM. Long-term assessments have shown no evidence of toxicity following repeated exposure, supporting the feasibility of sustained treatment. Clinical experience across other medical fields, including oncology and dermatology, similarly report a favorable safety profile. In neurological applications, adverse events have been infrequent and generally mild, most commonly consisting of headaches or scalp warmth.

Despite promising preclinical evidence and emerging clinical data, a significant gap remains in the literature regarding the systematic evaluation of the efficacy and safety of PBM on motor outcomes in PD [22,23,24,25]. Although individual studies have explored PBM applications across various neurological conditions, no systematic review has synthesized the available evidence from clinical trials that specifically examined PBM interventions in patients with PD. To address this critical knowledge gap, this study is, to the best of our knowledge, the first systematic review and meta-analysis to comprehensively analyze the current state of clinical evidence regarding PBM therapy for PD. As motor impairment represents the most established and clinically measurable manifestation of Parkinson’s disease, motor outcomes were selected as the primary focus of this initial review, including Movement Disorder Society-Unified Parkinson’s Disease Rating Scale Part III (MDS-UPDRS-III) scores, the Timed Up and Go (TUG) test, and the Ten-Meter Walk Test (TMWT), while also characterizing the safety profile of this therapeutic intervention in patients with PD. This review should be viewed as a first step within a broad evaluation of PBM in PD rather than as a comprehensive assessment of all its potential effects. Where sufficient data were available, a quantitative meta-analysis was performed to provide pooled estimates of treatment effect.

## 2. Materials and Methods

### 2.1. Study Design and Protocol Registration

This systematic review was conducted in accordance with the Preferred Reporting Items for Systematic Reviews and Meta-Analyses (PRISMA) guidelines [26]. The study protocol was prospectively registered in the International Prospective Register of Systematic Reviews (PROSPERO) under registration number CRD420261411269. The protocol prespecified the eligibility criteria and primary motor outcomes. MCID-based comparisons and additional sensitivity analyses were identified as exploratory analyses, as they were not explicitly included in the original protocol.

### 2.2. Eligibility and Exclusion Criteria

The study selection was based on the following inclusion criteria: (1) adult participants (≥18 years) with a clinical diagnosis of PD; (2) use of photobiomodulation (PBM), the preferred contemporary term, used throughout this review, regardless of the light source or delivery method, with the historical designation low-level light (or laser) therapy (LLLT) retained only where it appears in the search strategy and in the terminology of the source publications; (3) clinical trial design (randomized or non-randomized); and (4) English-language publications. No restrictions were placed on the duration of follow-up or publication. Studies were excluded if they were (1) reviews, commentaries, or editorials; (2) animal or preclinical studies; (3) observational studies; (4) case reports or case series; (5) qualitative studies; or (6) contained insufficient or non-extractable outcome data.

### 2.3. Search Strategy

A comprehensive search was conducted across five databases: PubMed, Embase, Web of Science, Google Scholar, and the Cochrane Library. Google Scholar was consulted only during the preliminary scoping of the literature to orient the research question before the protocol was completed; no study included in this review was identified or retrieved through it. It is listed here for transparency of the full search process rather than as a formal source of included records. Studies were identified based on the following criteria: (1) clinical trials on PBM therapy for PD, (2) English-language publications, and (3) articles published before 7 May 2026.

The search strategy employed a combination of keywords and Medical Subject Headings (MeSH) terms related to two main concepts: Parkinson’s disease (including terms such as “Parkinson*”, “PD,” “parkinsonism”) and photobiomodulation therapy (including “photobiomodulation,” “low-level light therapy,” “LLLT,” “red light therapy,” “near-infrared light,” “phototherapy”). Boolean operators (AND, OR) were used to appropriately combine the search terms. For each database, the search strategy was adapted to optimize retrieval while maintaining consistency across platforms. The complete search strategy for all databases is provided in Supplementary Table S1.

### 2.4. Study Selection and Data Extraction

The search results from all databases were imported into Rayyan [27] for systematic screening and reviewer blinding. Two independent reviewers (JVR and EPM) conducted title and abstract screening to assess the relevance of the study to the predefined inclusion criteria. Potentially eligible studies were included in the full-text review, as determined by both reviewers. During the full-text screening phase, both reviewers independently evaluated all articles to determine their final inclusion. Disagreements at any stage of the screening process were resolved through discussion between the two reviewers. Rayyan was used only as a reference-management and reviewer-blinding platform; no automated inclusion, exclusion, or machine-learning prediction feature was used at any stage, and all screening decisions were made independently by the two human reviewers. Records arising from the same trial were identified and linked, and the trial rather than the report was used as the unit of analysis. Where a trial had produced more than one publication, participant characteristics, safety data, and treatment exposure were extracted once only.

Data extraction was performed independently by two reviewers using a standardized data extraction sheet developed in Microsoft Excel and specifically designed to extract study characteristics from both randomized and non-randomized trials. Extracted data included study identifiers (first author, publication year, country of origin), methodological characteristics (study design, sample size, randomization method), participant demographics (age, sex, disease duration, Hoehn and Yahr stage, medication status), intervention parameters (device used, type of device, source of light, location of stimulations, wavelength used, number of diodes), intervention protocols (duration of sessions, frequency of sessions, duration of intervention, use of a combined intervention), outcome measures (primary and secondary motor outcomes with assessment tools and time points), and safety data, including all reported adverse events.

When key data were missing or unclear, the corresponding authors were contacted via email with specific requests for additional information.

### 2.5. Risk-of-Bias Assessment and Certainty of Evidence

Methodological quality and risk of bias were assessed using the Cochrane Risk-of-Bias 2.0 (RoB 2) tool, and the ROBINS-I tool was used for non-randomized studies [28,29]. Two reviewers independently assessed the risk of bias in each study (JVR and EPM). Each domain was rated as low risk, some concerns, or high risk of bias (RoB 2) or low, moderate, serious, or critical risk of bias (ROBINS-I). Discrepancies between reviewers were resolved through discussion to reach a consensus.

Certainty of evidence was assessed for each meta-analyzed outcome using the GRADE approach, considering risk of bias, inconsistency, indirectness, imprecision, and publication bias. As all studies contributing to the meta-analysis were randomized trials, assessment started at high certainty [30].

### 2.6. Data Synthesis and Analysis

To better assess the clinical relevance of these findings and to provide a more comprehensive evaluation of the effects of PBM, the results were compared against Minimal Clinically Important Difference (MCID) thresholds established in the literature. Each pooled estimate and its confidence limits were expressed as a multiple of the corresponding MCID to quantify how far the observed effect fell from the threshold of clinical importance. For MDS-UPDRS-III, a reduction of 3.25 points is the threshold for minimal clinically important improvement, while a reduction of 6.7 points defines moderate improvement [31]. In the original UPDRS Part III, a reduction of approximately 2.5 points is considered minimal, 5.2 points moderate, and 10.8 points large [32]. For the TUG test, a reduction of 3.5 s represents the MCID [33]. For the TMWT, an increase of 0.05 m/s in walking speed must be observed. Because TMWT reports completion time rather than walking speed, the speed-based MCID cannot be directly applied to the pooled time-based estimates [34].

Where sufficient data were available, random-effects meta-analyses were performed using the R package meta (version 7.0-0) [35]. Intervention and comparator group means, standard deviations, and sample sizes were extracted for each outcome. Meta-analyses used post-intervention values rather than change-from-baseline scores because change-score standard deviations and within-participant correlations were not consistently reported. The mean difference (MD) was calculated as the post-intervention mean in the PBM group minus that in the comparator group; negative values therefore favored PBM. Baseline values were extracted descriptively but were not used for covariate adjustment. Between-study heterogeneity was estimated using the restricted maximum-likelihood (REML) method, and the Hartung–Knapp–Sidik–Jonkman (HKSJ) adjustment was applied to confidence intervals. Statistical heterogeneity was quantified using I^2^.

For crossover studies, only data from the first randomized period were used when an independent-group comparison was available. Later periods were excluded to avoid repeated participant contributions and carryover effects; therefore, no within-participant correlation was required. Crossover studies without compatible data were summarized narratively. For multi-arm trials, only the prespecified PBM-versus-control comparison was included, while other intervention arms were excluded to avoid reuse of comparator participants [36].

A sensitivity analysis was prespecified to assess the influence of scale heterogeneity by excluding any study that used a non-standard version of MDS-UPDRS-III. An exploratory subgroup analysis was planned according to device architecture and wavelength. Estimates were pooled using the same random-effects model as the primary analyses, and differences between subgroups were assessed using a chi-square test based on the pooled subgroup estimates and their standard errors. Formal meta-regression on wavelength, fluence, or total radiant energy was not performed, as each meta-analysis contained between two and four studies, and total radiant energy was unreported in several trials. All subgroup analyses were post hoc and are reported as hypothesis-generating only. For studies not eligible for meta-analysis, Cohen’s d was calculated to characterize effect size (d > 0.5 moderate; d > 0.8 large) [37].

All studies contributing to the pooled estimates were randomized controlled trials (RCTs); the two non-randomized studies were not included in any meta-analysis and informed the narrative synthesis only. A post hoc sensitivity analysis restricted to trials at low overall risk of bias was therefore performed for MDS-UPDRS-III and TUG. It was not estimable for the TMWT, where only one contributing trial was rated low risk. Although the primary focus of this review was motor function, cognitive outcomes were extracted where reported, and a post hoc random-effects meta-analysis of the Montreal Cognitive Assessment (MoCA) was performed using the model specified above.

## 3. Results

### 3.1. Study Selection

**Figure 1:**
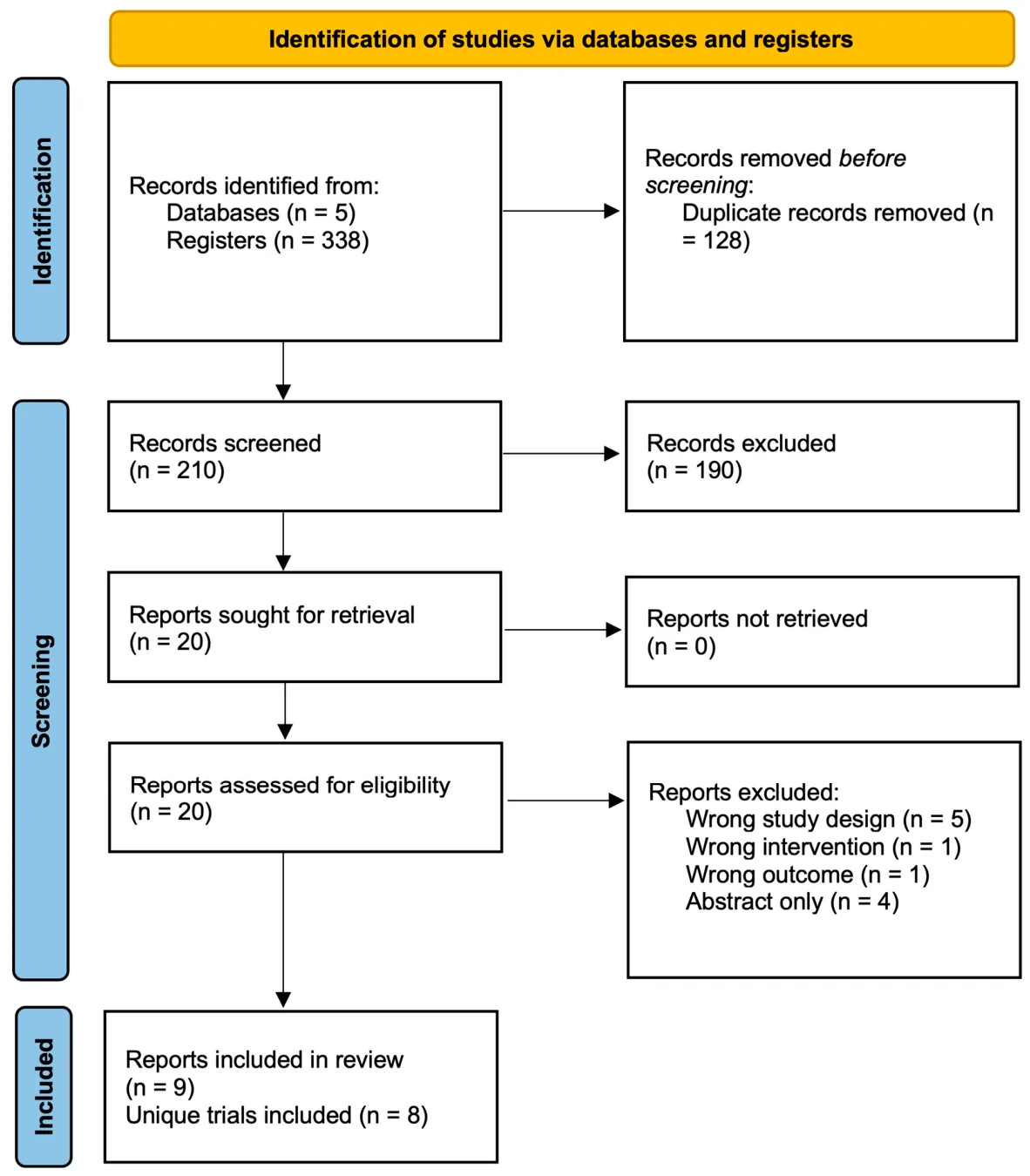
PRISMA flowchart.

The initial database search yielded 338 records. After removing duplicates (n = 128), 210 records were screened at the title and abstract levels. Of these, 190 were excluded based on the predefined criteria, leaving 20 full-text articles that were assessed for eligibility. Following a full-text review, 11 articles were excluded for the following reasons: wrong study design (n = 5), wrong intervention (n = 1), wrong outcome (n = 1), and abstract only (n = 4). The nine included articles corresponded to eight unique trials: McGee et al. 2023 and Herkes et al. 2023 are companion publications of a single randomized trial (ACTRN 12621001722886) [38,39], the former reporting a post hoc analysis of motor sub-scores from stage 1 and the latter the full feasibility report including the extension stage. The complete study selection process is illustrated in the PRISMA flow diagram (Figure 1).

### 3.2. Characteristics of Included Studies

The nine included reports corresponded to eight unique trials, comprising a total sample of 279 patients with PD, conducted between 2019 and 2025. Six trials employed randomized controlled designs: Battesha et al. 2025, Bullock-Saxton et al. 2021, Herkes et al. 2023, McGee et al. 2023, Saltmarche et al. 2025, Santos et al. 2019, and Santos et al. 2025 [38,39,40,41,42,43,44]. Among these, Saltmarche et al. 2025 used a double-blind three-stage crossover format—the largest trial to date—and Santos et al. 2025 employed a four-arm single-blind parallel-group design (control, exercise, PBM, and exercise + PBM) [39,42,44]. The remaining two studies utilized non-randomized designs: one open-label single-arm study and one waitlist-controlled prospective trial [23,45]. Geographically, two trials were conducted in Australia, two in Spain, one in Canada, one in Taiwan, and one in Jordan. Disease severity, as measured by the Hoehn and Yahr scale, ranged from stages 1–2 [43,44] to stages 2–3 or above across the remaining studies. All participants maintained functional independence. None of the nine included papers reported neurophysiological, neuroimaging, or molecular measures of target engagement. Specifically, no trial reported electroencephalography, TMS–EEG, functional MRI, positron emission tomography, single-photon emission computed tomography, near-infrared spectroscopy, or biofluid biomarkers. Reported outcomes were restricted to clinical rating scales and timed functional tests. The characteristics of the study population are presented in Table 1.

Helmet-based PBM devices designed for transcranial stimulation were used in three trials [23,38,39,42]. These systems employ LED light with wavelengths ranging from 810 to 904 nm, delivered in pulsed mode through multifocal arrays targeting more than 20 anatomical sites, including the temporal, frontal, and occipital regions, with some extending to intraoral applications. The remaining studies employed tabletop or handheld devices with interchangeable probes. Bullock-Saxton et al. 2021 applied pulsed laser stimulation at 904 nm to specific anatomical locations, including the temporal regions, intraoral sites, and the central sagittal suture [41]. Santos et al. 2019 and Santos et al. 2025 utilized 670 nm wavelength stimulation targeting bilateral temporal regions [43,44]. Battesha et al. 2025 employed transcranial PBM targeting the frontal and parietal regions at 810 nm in continuous-wave mode [40].

PBM radiant intensity, fluence, and total radiant energy showed significant variation across studies. Bullock-Saxton et al. 2021 reported a radiant intensity of 306 mW/cm^2^, a fluence of 10.2 J/cm^2^, and a total radiant energy of 42 J per session, while Hong et al. 2021 applied a much lower radiant intensity (6 mW/cm^2^), with an estimated fluence of ~10.8 J/cm^2^, but did not report total energy [41,45]. Santos et al. 2019 delivered 60 mW/cm^2^ with a fluence of 21.6 J/cm^2^, without reporting total radiant energy. In contrast, helmet-based protocols delivered substantially higher total energies, with Herkes et al. 2023 and McGee et al. 2023 reporting approximately 1137.6 J per session, though radiant intensity and fluence were not specified [38,39,43]. Overall, dosimetric reporting was inconsistent, and total radiant energy per session varied by more than an order of magnitude. The complete set of study parameters is presented in Table 2.

Studies demonstrated considerable variation in treatment protocols. Session durations ranged from approximately 9 min to 30 min, with Herkes et al. 2023 and McGee et al. 2023 employing 24 min sessions [38,39,43,45]. The total number of sessions also varied substantially: Santos et al. 2019 provided 18 sessions, Bullock-Saxton et al. 2021 and Liebert et al. 2021 provided 24 and 36 sessions, respectively, Saltmarche et al. 2025 provided 36 sessions across three crossover stages, McGee et al. 2023 delivered 72 sessions, and Hong et al. 2021 implemented 10 sessions with unreported individual session durations [23,38,41,42,43,45]. Santos et al. 2019 incorporated a combined intervention approach, supplementing PBM with hydrogen water administration [43]. All other studies evaluated PBM as a standalone intervention or, in the case of Santos et al. 2025, also assessed PBM in combination with exercise in separate trial arms [44].

Most studies were conducted in research center environments with researcher administration. Santos et al. 2019 uniquely implemented a hybrid approach combining research center visits with home-based sessions, involving both researchers and patients in protocol administration [43]. Most studies conducted two or more follow-up assessments to evaluate treatment persistence. The heterogeneity in protocol parameters reflects the early-stage nature of PBM research in PD. The complete study protocol is presented in Table 3, and the motor and non-motor tests assessed in each study are listed in Table 4. To ease clinical interpretation, pooled mean differences were compared with MCID thresholds in Supplementary Table S3.

### 3.3. Risk of Bias in Studies

**Figure 2:**
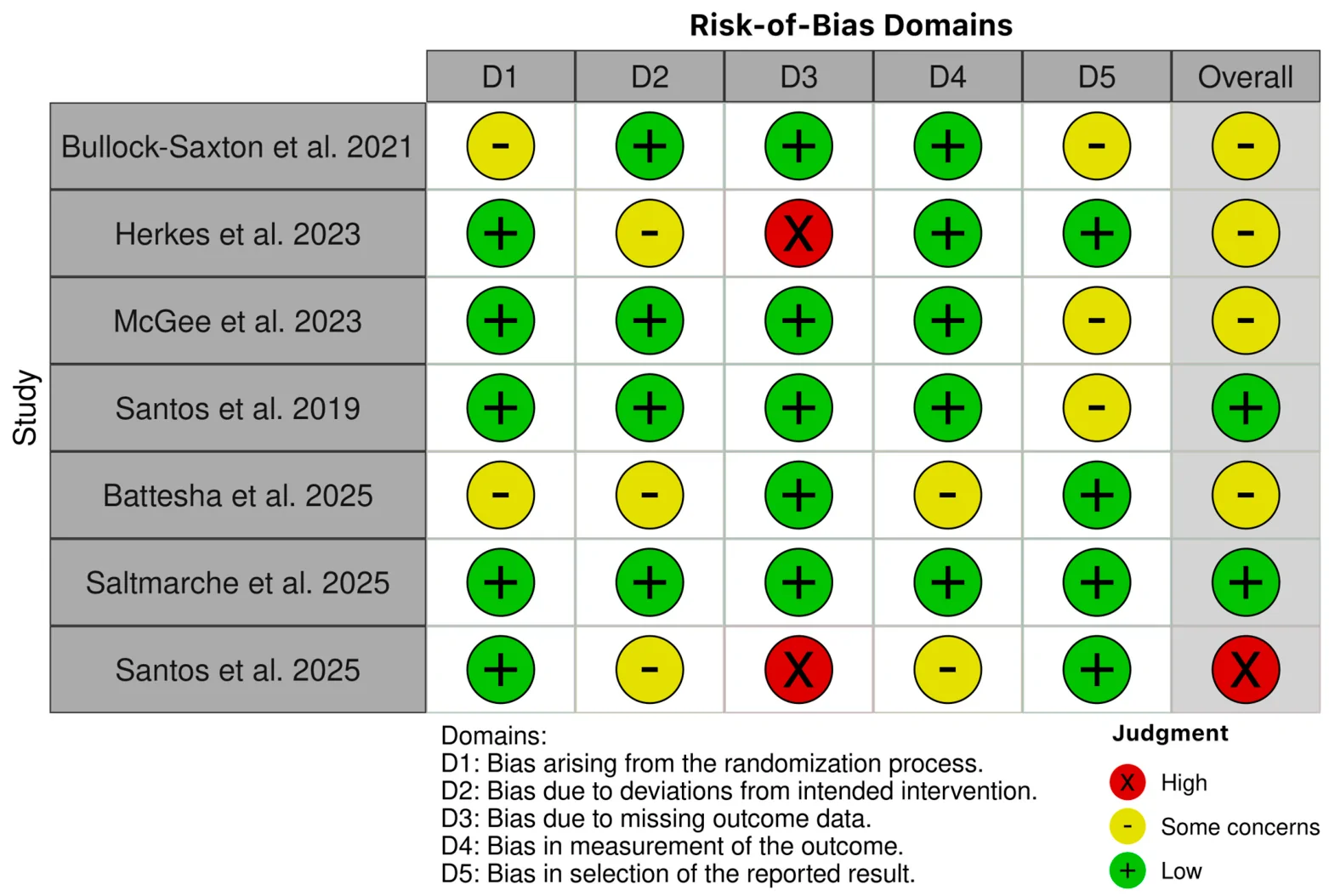
Risk-of-bias assessment of the included RCTs using the RoB 2 tool, showing domain-level and overall judgments across five methodological domains of Bullock-Saxton et al. 2021, Herkes et al. 2023, McGee et al. 2023, Santos et al. 2019, Battesha et al. 2025, Saltmarche et al. 2025, Santos et al. 2025 [38,39,40,41,42,43,44].

Risk of bias was assessed at the individual level, as specified by RoB 2, rather than by each trial. The nine included publications were assessed using RoB 2. Overall judgments were low risk for Santos et al. 2019 and Saltmarche et al. 2025 [42,43]; some concerns for Bullock-Saxton et al. 2021, Battesha et al. 2025, and McGee et al. 2023 [40,41]; and high risk for Herkes et al. 2023 and Santos et al. 2025 [38,39,44]. The high overall risk for Herkes et al. 2023 and Santos 2025 was driven by domain 3, concerning bias due to missing outcome data [39,44]. McGee et al. 2023 and Herkes et al. 2023 report different results from the same randomized trial (ACTRN 12621001722886) and were therefore assessed separately [38,39]. Concerns regarding domain 5, focused on the selection of reported results, were identified in Bullock-Saxton et al. 2021, McGee et al. 2023, and Santos et al. 2019 [38,41,43]. Battesha et al. 2025 identified several concerns in domains 1, 2, and 4 [40]. These concerns involve the inadequate documentation of randomization and allocation procedures, deviations from the intended intervention, and outcome-measurement procedures. The complete domain-level judgments and their rationales are presented in Figure 2 and Supplementary Table S4 [37,38,39,40,41,42,43].

**Figure 3:**
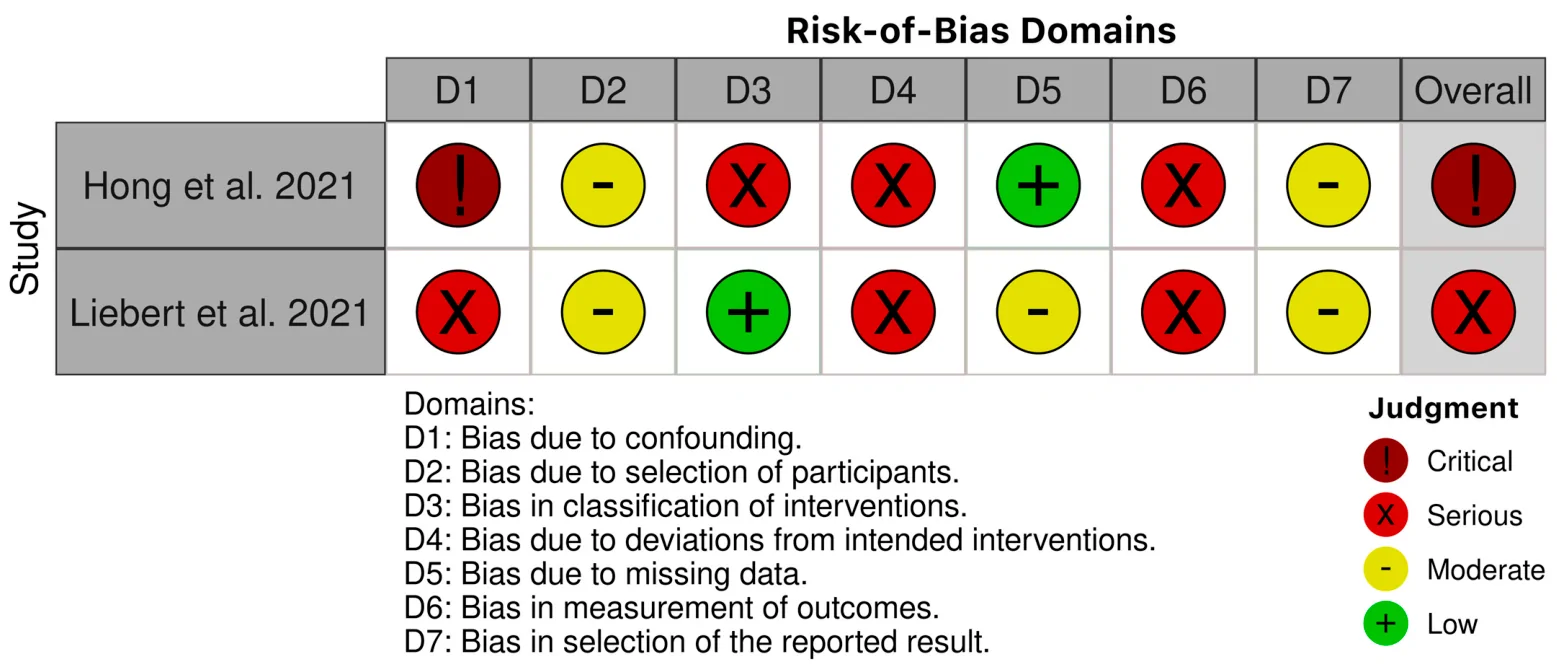
Risk-of-bias assessment of non-randomized clinical trials using the ROBINS-I tool across all seven domains of Hong et al. 2021 and Liebert et al. 2021 [23,45].

The two non-randomized trials were assessed using the ROBINS-I tool. Hong et al. 2021 [45] received a “critical” risk-of-bias rating, the highest level of concern and indicating serious flaws that significantly compromise the validity of the study findings, while Liebert et al. 2021 [23] was assessed as having “serious” risk-of-bias concerns according to the ROBINS-I tool. The complete domain-level judgments and their rationales are presented in Figure 3 and Supplementary Table S5.

Overall, the methodological limitations varied across studies and domains, ranging from low risk to high or critical risk of bias. These limitations reduce the certainty with which the clinical effects of PBM can be interpreted and emphasize the need for larger, rigorously designed, preregistered randomized trials with complete outcome reporting.

### 3.4. Outcomes Results of Individual Studies

Several studies reported MDS-UPDRS-III or UPDRS-III motor scores. McGee et al. 2023 found no statistically significant between-group difference in total MDS-UPDRS-III scores [38]. The active group exhibited a within-group change of −4.90 points, exceeding the MCID of 3.25 points; however, the sham group demonstrated a larger within-group reduction of −5.53 points. The between-group post-treatment difference favored PBM (MD = −4.02 points, 95% CI: −11.40 to 3.36) but was not statistically significant and likely reflects baseline imbalance rather than a true treatment effect, with an overall negligible between-group effect size (Cohen’s d = 0.06). Santos et al. 2019 reported a mean reduction of only 1 point in the intervention group, which did not meet the MCID threshold, while the sham group showed no change (between-group MD = 1.00 point, 95% CI: −1.65 to 3.65; Cohen’s d = 0.22) [43]. Herkes et al. 2023 observed no significant between-group differences at any assessment time point [39]. Although a mean difference of 3.9 points was noted after Stage 1, exceeding the MCID, this effect was transient—Stage 2 showed a reversal with a mean difference of −3.1 points (95% CI: 2.7 to −10.6)—yielding a negligible overall impact (Cohen’s d = 0.17). Bullock-Saxton et al. 2021 and Liebert et al. 2021 administered MDS-UPDRS-III but did not report extractable results [23,41]. Hong et al. 2021 cited UPDRS-III but provided only a single incomplete plot [45].

Saltmarche et al. 2025 conducted the largest RCT to date (n = 63), employing a three-stage double-blind crossover design [42]. No statistically significant between-group differences in MDS-UPDRS-III scores were observed after Stage 1 or Stage 2. After Stage 3, participants who continued active treatment showed significant improvement compared with non-continuers (between-group MD = −3.30 points, 95% CI: −9.00 to 2.40), suggesting a possible cumulative effect requiring longer treatment duration. MDS-UPDRS Part II was also significantly improved, and the MDS-UPDRS total score approached significance (*p* = 0.062). No significant between-group TUG differences were observed, although within-group improvements were noted in both groups.

Santos et al. 2025 employed a four-arm parallel-group RCT (control, exercise, PBM, exercise + PBM) in patients with mild PD (H and Y stages 1–2, n = 53) [44]. For the primary comparison of PBM versus control, post-intervention MDS-UPDRS-III scores were 9.80 ± 7.50 (PBM, n = 13) versus 13.20 ± 10.20 (control, n = 15), corresponding to a between-group MD of −3.40 points (95% CI: −9.98 to 3.18), which was not statistically significant and did not exceed the MCID threshold. Post-intervention TUG was 9.1 ± 3.4 s (PBM) versus 10.2 ± 3.3 s (control), also non-significant (MD = −1.10 s, 95% CI: −3.59 to 1.39).

Battesha et al. 2025 (n = 38; Jordan) compared transcranial PBM with conventional physical therapy over 12 weeks [40]. Statistically significant improvements in postural stability and functional activity level were observed in the PBM group relative to the control (*p* < 0.05). This study did not report MDS-UPDRS-III, TUG, or TMWT and could therefore not be included in the quantitative meta-analysis.

TUG was evaluated in multiple studies. Liebert et al. 2021 found a mean difference of −1.21 s in standard TUG (Cohen’s d = 0.41), and Santos et al. 2019 reported a between-group MD of −2.20 s (95% CI: −4.96 to 0.56; Cohen’s d = 0.28) [23,43]. For TUG motor performance, Bullock-Saxton et al. 2021 demonstrated small improvements in both groups (intervention: −1.2 s; sham: −0.3 s; Cohen’s d = 0.22), and Liebert et al. 2021 reported −0.75 s for TUG motor (Cohen’s d = 0.25) [23,41]. Across all studies, no TUG outcome achieved the 3.5 s MCID threshold or reached statistical significance.

Walking speed was evaluated using the TMWT in two studies. Santos et al. 2019 reported a statistically significant reduction of 0.6 s in walking time in the PBM group, with no change in the sham group (Cohen’s d = 0.626, *p* < 0.01) [43]; direct comparison to the speed-based MCID (0.05 m/s) was not possible with a time-based metric. Santos et al. 2025 showed a post-intervention TMWT of 3.9 ± 1.1 s (PBM) versus 4.4 ± 1.4 s (control) [44]; the difference was numerically small and non-significant. Liebert et al. 2021 observed statistically significant gait speed improvements at 12 weeks (*p* = 0.03) that were not sustained at the 40-week follow-up, suggesting a lack of long-term benefit without continued intervention [23].

Some studies assessed fine motor dexterity using the Nine-Hole Peg Test. Liebert et al. 2021 found no statistically significant improvements (*p* > 0.05), while Bullock-Saxton et al. 2021 reported changes meeting the minimum clinically important difference threshold, although specific values and statistical significance were not clearly described [23,41].

### 3.5. Exploratory Quantitative Synthesis

All pooled estimates come from randomized controlled trials, with overall RoB 2 judgments ranging from low (Santos et al. 2019, Saltmarche et al. 2025) [42,43] through some concerns (McGee et al. 2023) [38] to high (Santos et al. 2025) [44]; the two non-randomized studies contributed only to the narrative synthesis.

Four main meta-analyses were conducted: a primary analysis for MDS-UPDRS-III (four RCTs: McGee et al. 2023, Santos et al. 2019, Saltmarche et al. 2025, Santos et al. 2025) [38,42,43,44], an analysis for TUG (three RCTs: Santos et al. 2019, Saltmarche et al. 2025, Santos et al. 2025) [42,43,44], a sensitivity analysis for MDS-UPDRS-III excluding McGee et al. 2023 [38]—which used a modified 37-item scale—to assess the influence of scale heterogeneity, and an exploratory meta-analysis of walking speed (TMWT; two RCTs: Santos et al. 2019 [43] and Santos et al. 2025 [44]).

**Figure 4:**
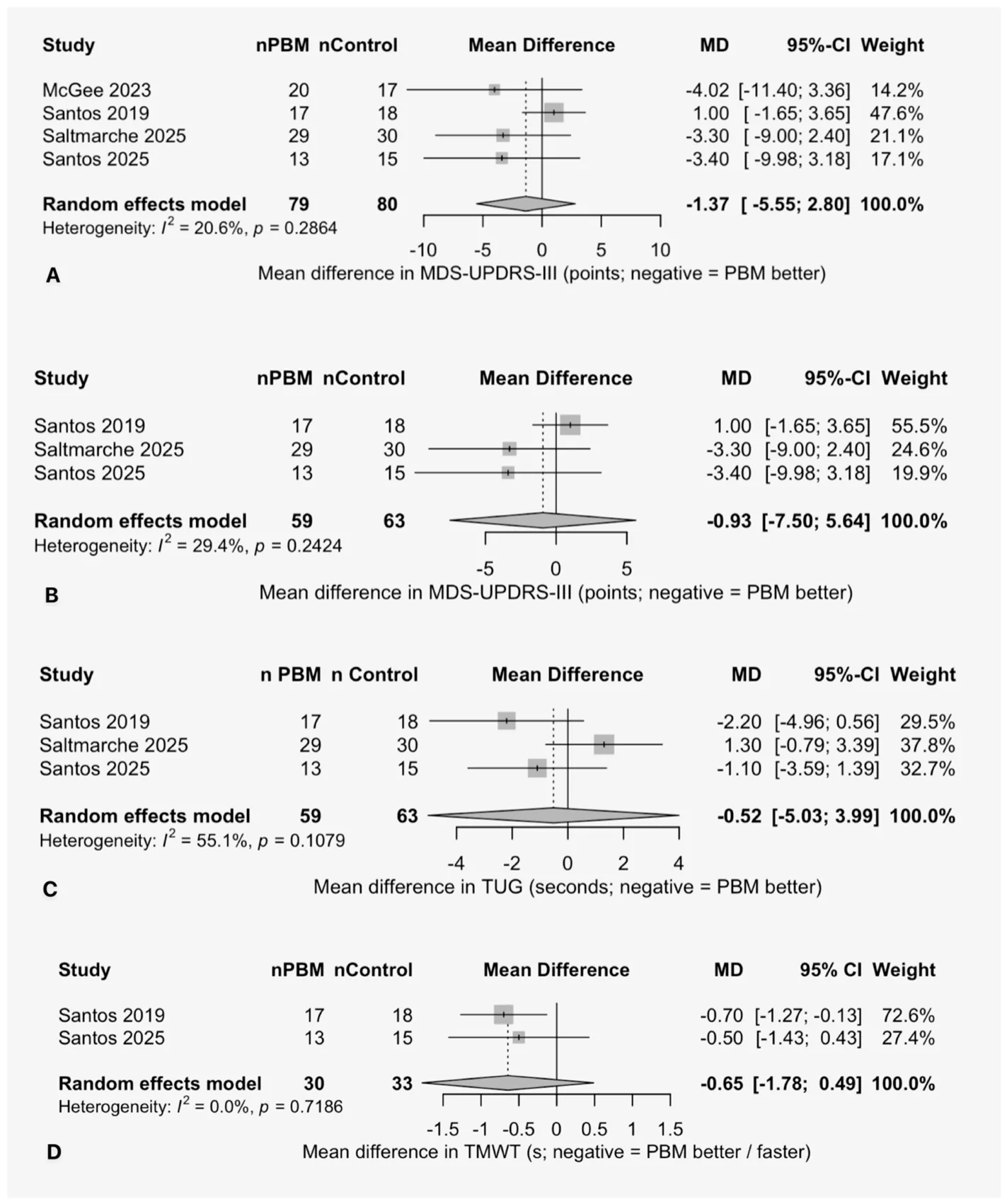
(**A**) Forest plot of the effect of photobiomodulation on MDS-UPDRS-III scores. (**B**) Sensitivity analysis—forest plot of MDS-UPDRS-III excluding McGee et al. 2023 [38]. (**C**) Forest plot of the effect of photobiomodulation on Timed Up and Go performance. (**D**) Forest plot of the effect of photobiomodulation on Ten-Meter Walk Test (TMWT).

Meta-analysis of the four MDS-UPDRS-III RCTs found no statistically significant pooled effect of PBM on motor scores (MD = −1.37 points, 95% CI: −5.55 to 2.80; I^2^ = 20.6%; Figure 4A). Individual study MDs ranged from −4.02 points (McGee et al. 2023) [38] to +1.00 point (Santos et al. 2019) [43], and none exceeded the between-group MCID of 3.25 points. The low heterogeneity (I^2^ = 20.6%, *p* = 0.29) indicates broadly consistent null findings across trials.

The sensitivity analysis excluding McGee et al. 2023 [38] yielded a comparable result (MD = −0.93 points, 95% CI: −7.50 to 5.64; I^2^ = 29.4%; Figure 4B), confirming that the primary finding was not driven by scale heterogeneity.

Meta-analysis of the three TUG RCTs likewise showed no significant pooled effect (MD = −0.52 s, 95% CI: −5.03 to 3.99; I^2^ = 55.1%; Figure 4C). The moderate heterogeneity reflects opposing directions of effect: Santos et al. 2019 [43] (MD = −2.20 s) and Santos et al. 2025 [44] (MD = −1.10 s) favored PBM, while Saltmarche et al. 2025 [42] (MD = +1.30 s) marginally favored the control condition, suggesting genuine between-trial variability rather than methodological artifact. No study met the 3.5 s MCID threshold in the between-group comparison.

Meta-analysis of walking speed (TMWT) yielded a pooled MD of −0.65 s (95% CI: −1.78 to 0.49; I^2^ = 0.0%; Figure 4D) in favor of PBM, which did not reach statistical significance. Both studies showed directionally consistent MDs (−0.70 s and −0.50 s, respectively) with no heterogeneity [43,44], though the conservative HKSJ correction at k = 2 substantially widens the confidence interval. As both studies originate from the same research group, this analysis is an exploratory analysis only.

In individual studies not included in the meta-analysis, effect sizes ranged from negligible (Cohen’s d = 0.03–0.06) to small-to-moderate (d = 0.41), with no individual estimate reaching its respective MCID threshold for a clinically meaningful treatment signal. Funnel plots for both primary meta-analyses are presented in Supplementary Figures S1 and S2; formal asymmetry testing was not performed given the small number of contributing studies.

An exploratory subgroup analysis by device/wavelength class was performed (Supplementary Table S6 and Supplementary Figure S3). For MDS-UPDRS-III, the NIR-helmet subgroup yielded MD = −3.57 points (95% CI −7.99 to +0.86; I^2^ = 0%), a point estimate exceeding the 3.25-point MCID although its confidence interval crossed the null. The red-local-probe subgroup was essentially null (MD = −0.13), and the subgroup difference was not significant (*p* = 0.078). For TUG, the order was reversed, with red local probes numerically favored (MD = −1.59 s) against the single NIR-helmet trial (MD = +1.30 s; *p* = 0.016). No comparison was estimable for TMWT, as both contributing trials used red local probes. These are unstable results, as the direction of effect reverses between outcomes, with each subgroup containing only one to two trials, and the device architecture is confounded with wavelength.

A post hoc sensitivity analysis restricted to the two trials at low overall risk of bias (Santos et al. 2019; Saltmarche et al. 2025) [42,43] did not alter the direction or the interpretation of the pooled estimates. For MDS-UPDRS-III, the restricted estimate was MD = −0.38 points (95% CI −25.89 to 25.13; I^2^ = 44.4%; k = 2), and for TUG, it was MD = −0.33 s (95% CI −22.51 to 21.85; I^2^ = 74.5%; k = 2). In both cases, the point estimate remained small and non-significant, but the confidence intervals widened substantially, reflecting the conservative HKSJ adjustment applied at k = 2 (one degree of freedom). These analyses therefore confirm that restricting the evidence base to the lowest-risk trials does not reveal a treatment effect obscured by the inclusion of higher-risk studies; they also illustrate how little precision remains once the evidence base is narrowed.

Two RCTs reported extractable MoCA data (Bullock-Saxton et al. 2021; Saltmarche et al. 2025) [41,42]. Pooled analysis showed no significant effect of PBM on cognitive performance (MD = −0.94 points, 95% CI −3.78 to +1.90; I^2^ = 0.0%; *p* = 0.149; k = 2). As higher MoCA scores denote better cognition, this estimate numerically favors the control condition, and its magnitude lies below commonly cited MCID thresholds for the MoCA in PD. No included trial reported extractable data on mood, sleep, or quality of life, and these domains could not be synthesized. This analysis was post hoc and exploratory. Funnel plots (Supplementary Figures S1 and S2) were inspected. However, with four and three contributing studies, respectively, these plots have little power to detect small-study effects, and formal asymmetry testing (e.g., Egger’s test) was not performed, as it is not recommended below approximately ten studies.

### 3.6. Safety and Tolerability

PBM demonstrated a favorable safety profile and was generally well tolerated across all eight included trials. McGee et al. 2023 [38] reported three device-related adverse events in the active treatment group: temporary leg weakness in one participant, decreased right-hand fine motor skills in another, and minor dizziness during a session in a third participant. All events resolved spontaneously without intervention. No severe adverse events were observed in any of the included studies. Treatment adherence consistently exceeded 90% in all trials reporting this outcome, indicating the feasibility of PBM intervention and patient acceptance.

### 3.7. Certainty of Evidence

Certainty of evidence was rated very low for all three motor outcomes (Table 5). Assessment began at high certainty, as all contributing studies were randomized trials. All three outcomes were downgraded one level for risk of bias, reflecting trials that presented low-to-high overall risk on RoB 2, and two further levels for imprecision, given the small number of trials and confidence intervals extending beyond the MCID in both directions. TUG was additionally downgraded for inconsistency (I^2^ = 55.1%), reflecting opposing directions of effect across trials. Publication bias could not be assessed for any outcome, as funnel plots and asymmetry testing have essentially no power with two to four studies.

## 4. Discussion

### 4.1. Summary of Findings

This systematic review analyzed eight clinical trials that investigated PBM for motor outcomes in PD, complemented by a random-effects meta-analysis where data permitted. Neither the meta-analysis of MDS-UPDRS-III scores and TUG performance nor the exploratory meta-analysis of walking speed (TMWT) reached statistical significance, and no effect exceeded its respective MCID threshold. In individual studies, numerical improvements were observed across various motor assessments, but these predominantly fell below thresholds for clinical significance. Effect sizes calculated using Cohen’s d ranged from negligible (d = 0.03 to 0.06) to small-to-moderate (d = 0.41), with only the TMWT showing a moderate effect (d = 0.626) in one study. These patterns indicate that PBM, as currently tested, does not have reliable evidence of benefit for motor dysfunction in PD. Formal analysis using GRADE rated the certainty of evidence as very low for all three outcomes, downgraded mainly for imprecision and for risk of bias among the contributing trials. Overall, RoB 2 judgments among the included RCTs ranged from low to high risk, with concerns regarding selective reporting and missing outcome data, while the two non-randomized trials were rated “serious” or “critical” across multiple domains. This very low certainty is why these results should be read as inconclusive rather than negative.

These findings must be contextualized within the broader therapeutic landscape of PD. Systematic reviews and meta-analyses of deep brain stimulation (DBS) consistently demonstrate robust and reproducible benefits, with RCTs reporting improvements of up to 50% in MDS-UPDRS-III scores. Acute administration of levodopa typically produces a 20–30% reduction in MDS-UPDRS-III scores. The negligible-to-small effect sizes observed with PBM (Cohen’s d = 0.03 to 0.41) fall far below these established benchmarks. Although PBM may produce modest improvements in select motor measures such as walking speed, the clinical significance of these effects remains uncertain when compared with the magnitude of benefits achieved with established treatments.

Changes in MDS-UPDRS-III exceeding the MCID were reported only as within-group effects in McGee et al. 2023 and Herkes et al. 2023 [38,39], where comparable improvements occurred in sham groups or effects reversed over time. Santos et al. 2019 [43] did not reach the MCID for MDS-UPDRS-III, and no study achieved the MCID for TUG. The transient walking speed improvement observed in Santos et al. 2019 was not sustained in Liebert et al. 2021 [23,43], limiting its clinical interpretation.

Moreover, a lack of consistent correlation was also identified between PBM dosimetric parameters and clinical outcomes. Helmet-based interventions delivering the highest total radiant energy—approximately 1137 J per session, as reported by McGee et al. 2023 and Herkes et al. 2023 [38,39]—did not demonstrate superior motor improvements compared with lower-energy protocols. Only within these high-energy groups did MCID-exceeding changes occur, and they were transient or mirrored by sham conditions. Lower-energy approaches, such as those employed by Santos et al. 2019 and Bullock-Saxton et al. 2021 [41,43], also proved ineffective in producing consistent or durable motor benefits. The isolated gait improvement observed in Santos et al. 2019 [43] was not replicated in trials with higher energy delivery, arguing against a simple dose–response relationship. The lack of statistically significant pooled effects should not be interpreted as definitive evidence of no treatment effect.

### 4.2. Comparative Analysis Across Neurological Conditions

The consistently negative results in PD differ from the positive outcomes seen in PD animal studies and in human trials of other neurological diseases, raising questions about whether PBM works differently across conditions. In animal models, PBM has shown clear benefits, such as attenuation of neurodegeneration, improvements in locomotor activity, attenuation of neuroinflammation, and preservation of striatal dopaminergic terminals. However, these successes have not been translated into PD clinical trials, highlighting a major challenge in research: animal models may not fully capture the complex and progressive nature of human PD [46]. A recent systematic review of 35 transcranial PBM studies found that 29 (82.9%) showed positive outcomes, including all nine trials in participants with subjective memory complaints, mild cognitive impairment, or dementia [47].

Together, these findings highlight a differential response pattern that raises several possibilities: (1) acute or subacute conditions could be more responsive than chronic PD in patients; (2) the pathophysiology of PD may be inherently less amenable to PBM; (3) current PBM targets may face mechanistic limitations that restrict their efficacy; or (4) motor outcomes in PD may be intrinsically more difficult to modulate through cortical stimulation alone, given their dependence on intact cortico–subcortical–spinal feedback circuits—including the corticostriatal, thalamocortical, and corticospinal pathways—whose dysfunction in PD extends well beyond cortical processing.

In contrast, non-motor outcomes, particularly cognitive function, may represent more tractable targets for transcranial PBM. Cognitive processes such as executive function, attention, and working memory are predominantly orchestrated by cortical and prefrontal networks that lie within the range of photonic energy achievable under current transcranial protocols [48]. Aligned with this, functional near-infrared spectroscopy studies have demonstrated that transcranial PBM induces measurable increases in prefrontal cortical oxygenation that correlate directly with cognitive improvements, providing mechanistic credibility for the differential response pattern observed across conditions [49]. It should be noted, however, that the pooled MoCA data available in the present review did not support a cognitive benefit (Section 3.5); therefore, this hypothesis remains to be tested in trials designed with non-motor endpoints as primary outcomes.

### 4.3. Mechanistic Limitations and Target Engagement

Although the leading hypothesis suggests that near-infrared light enhances mitochondrial function via cytochrome c oxidase activation and thereby modulates dopaminergic circuits, none of the available clinical trials have incorporated biomarkers to confirm biological engagement [25]. Future PBM trials could incorporate quantitative susceptibility mapping (QSM) as an exploratory mechanistic and target-engagement outcome. QSM may provide information about regional magnetic susceptibility and iron-associated pathophysiology in Parkinson’s disease. However, magnetic susceptibility is not a direct or entirely specific measure of iron metabolism [50]. This issue is particularly relevant because PBM protocols estimate that less than 0.2% of incident photons reach the cortical layers under conventional transcranial conditions, while tissue-optics data demonstrate substantial attenuation as light passes through the scalp and skull [43,51,52]. Human cadaver studies indicate that penetration of 830 nm light varies by anatomical region, ranging from approximately 0.9% in the temporal region to 11.7% in the occipital region, while transmission of 810 nm light through the human skull has been reported at approximately 4%. Ex vivo models further suggest that between 0.45% and 2.9% of 810–980 nm light may penetrate combined scalp, skull, and superficial brain tissue layers [53]. Although near-infrared wavelengths (808–830 nm) consistently demonstrate greater penetration than visible red wavelengths (633–660 nm), the proportion of incident energy reaching cortical tissue remains limited, and the intracerebral power density required to induce biologically meaningful modulation has not been definitively established [54,55].

This attenuation becomes particularly relevant when considering deeper structures central to PD’s pathophysiology, such as the substantia nigra within the midbrain. The extent to which sufficient photonic energy reaches these dopaminergic nuclei under conventional helmet-based LED arrays remains uncertain. Alternative delivery approaches, including intranasal and intraoral PBM used in some included trials, may offer more anatomically direct pathways to ventral brain regions. The intranasal route may expose the olfactory bulb and basal forebrain, while intraoral delivery could potentially access ventral brainstem regions using wavelengths in the 800–900 nm range. Nevertheless, no human study has quantified intracerebral photon density at these subcortical targets or demonstrated direct dopaminergic circuit engagement.

**Figure 5:**
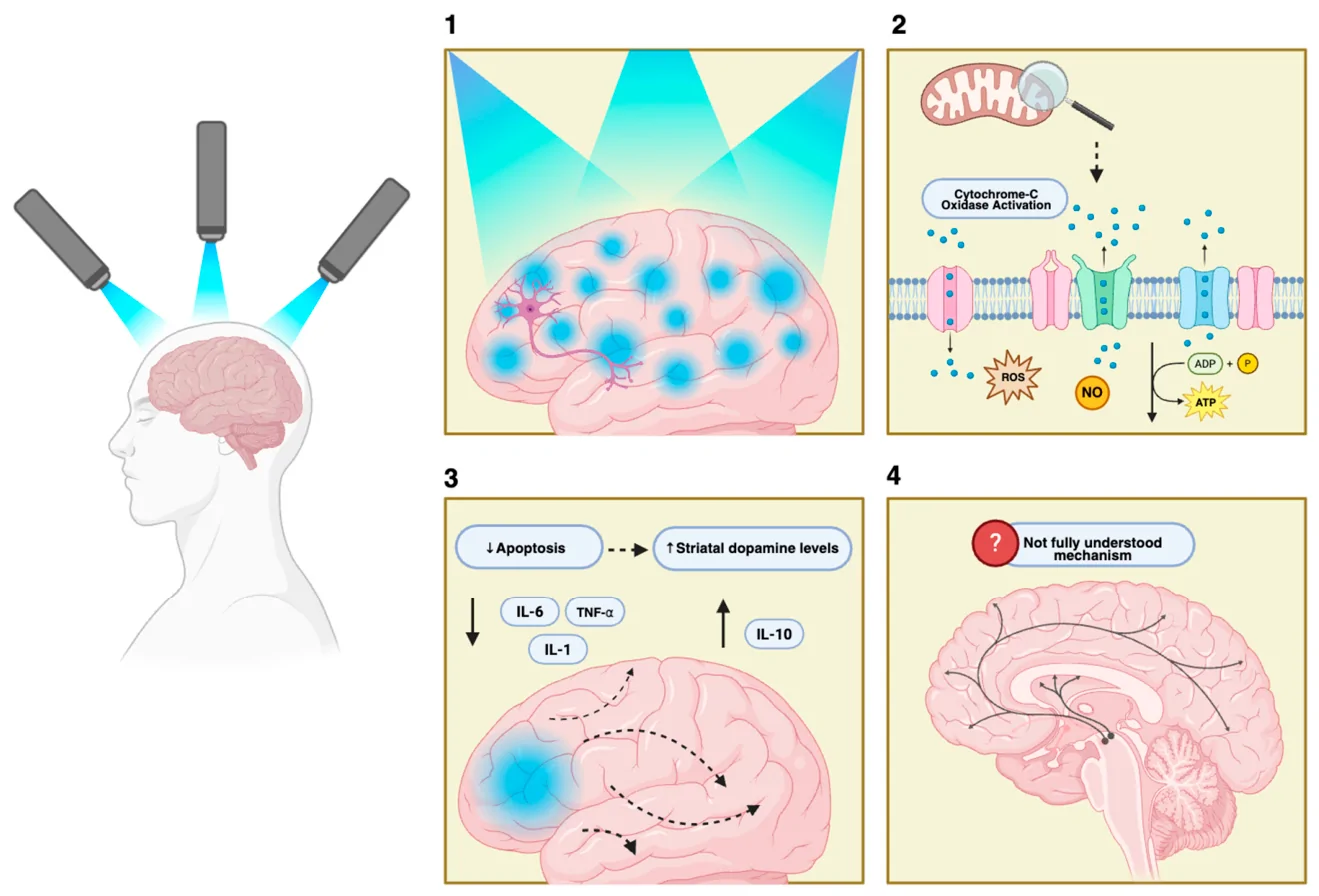
Proposed effects of PBM on dopaminergic pathways. Conceptual illustration created by the authors.

From a systems neuroscience perspective (Figure 5), PBM applied to cortical regions (e.g., motor and prefrontal cortices) could theoretically modulate distributed dopaminergic networks via top-down pathways [56]. For instance, cortical stimulation might influence the corticostriatal–thalamocortical loop, indirectly enhancing striatal dopamine tone through glutamatergic modulation or improved mitochondrial efficiency within cortical neurons projecting to the basal ganglia [57]. However, this remains speculative, and without concurrent neurophysiological or molecular data, it is impossible to determine whether such circuits are being effectively modulated in vivo.

This mechanistic uncertainty is likely a major contributor to the consistently null or marginal clinical effects observed across trials. If photonic energy is not reaching relevant brain targets—or if it is engaging tissue but not the disease-relevant circuits—then even optimal dosing schedules will fail to produce meaningful clinical outcomes. Future studies must therefore incorporate target engagement biomarkers to empirically validate the assumed mechanisms and optimize stimulation parameters accordingly.

Future trials should incorporate measures matched to the mechanism under test. For example, near-infrared spectroscopy or diffuse optical measurement can verify that photons reach cortical tissue at the intended irradiance; TMS–EEG or resting-state EEG can index cortical excitability and network engagement; task-based or resting-state functional MRI can assess corticostriatal circuit effects; and dopaminergic imaging (DaT-SPECT or PET) can be used where disease modification is claimed. Without such measures, it remains close to impossible to distinguish insufficient photonic delivery from an absence of biological effect, a distinction on which the interpretation of every trial in this review depends.

### 4.4. The Possible Role of the Placebo Effect and Sham Selection

Another critical source of bias in PBM trials for PD relates to the robust placebo response commonly observed in PD motor symptoms and the methodological variability in sham design. PD is characterized by pronounced dopaminergic placebo responsiveness, particularly in early stages of the disease. Placebo-induced dopamine release in the striatum has been well documented through PET imaging studies, with effects robust enough to produce measurable motor improvements [58,59]. This makes PD trials especially vulnerable to placebo-induced outcome inflation, particularly for subjective or semi-objective measures such as the MDS-UPDRS-III and TUG tests.

MCID-based interpretation corroborates the placebo contribution. In McGee et al. 2023 [38], the sham group exhibited a larger within-group reduction (−5.53 points) than the active PBM group (−4.90 points), with both exceeding the MCID threshold of 3.25 points; however, the between-group difference was negligible and non-significant (MD = −4.02 points, 95% CI: −11.40 to 3.36). The presence of MCID-level within-group improvements without consistent between-group differentiation is characteristic of placebo-responsive conditions such as PD. 

To address these limitations, future trials should (1) implement triple-masked designs, ensuring participants, investigators, and outcome assessors are blinded; (2) use non-illuminating shams or optically matched but biologically inert wavelengths verified through dosimetry modeling; (3) quantify and report expectancy scores and blinding integrity, ideally with questionnaires measuring perceived treatment allocation; and (4) incorporate objective digital biomarkers (e.g., gait analysis, wearable accelerometry) that are less susceptible to placebo modulation.

### 4.5. Ongoing Studies and Future Perspectives

Several ongoing clinical trials may provide additional data in the coming years. The EvNIR study (NCT04261569), which employs intracranial near-infrared light in patients with de novo PD over four years, represents the most ambitious PBM trial to date. Preliminary data from this study have suggested potential disease-modifying effects based on motor score improvements and dopamine transporter imaging findings, although these results require independent validation in larger cohorts with longer follow-up periods. Long-term follow-up studies, such as the 5-year extension of the Liebert cohort [60], demonstrate sustained improvements in some participants continuing home-based therapy, although the absence of control groups limits the interpretation of these findings.

### 4.6. Limitations

This systematic review has some limitations that should be acknowledged. The small number of included trials (n = 8; 279 patients) with modest individual sample sizes limited statistical power, although the consistency of null findings across studies strengthens our conclusions. One trial contributed two publications; treating individual articles as the unit of analysis would have inflated the size of the evidence. Heterogeneity in study design, intervention protocols, and outcome measures restricted the scope of quantitative synthesis; meta-analysis was feasible only for MDS-UPDRS-III (four studies), TUG (three studies) and walking speed (two studies), and the pooled estimates should be interpreted considering the small number of contributing studies and residual clinical heterogeneity. Restricting the analysis to trials at low overall risk of bias did not change the direction or magnitude of the pooled estimates, indicating that the null findings are not attributable to the inclusion of higher-risk trials. An exploratory subgroup analysis by device/wavelength class was undertaken but must be regarded as unstable: the direction of effect reversed between MDS-UPDRS-III and TUG, each subgroup contained only one to two trials, and device architecture was fully confounded with wavelength. Formal meta-regression across dosimetric parameters was precluded by the small number of studies and by incomplete reporting of total radiant energy. Consequently, potential parameter-specific effects remain uncertain. None of the included trials incorporated biomarkers of target engagement, making it impossible to determine whether null clinical results reflect insufficient photonic delivery, an absence of biological effect, or both. Publication bias may have influenced our findings, although the predominance of null results in the included literature argues against systematic suppression of negative data. The short treatment durations and follow-up periods in most studies limit assessment of longer-term effects. These limitations are reflected in the GRADE assessment: certainty of evidence was very low for every meta-analyzed outcome, meaning that the true effect of PBM on motor function in PD may differ substantially from the pooled estimates reported here. Two statistical caveats require emphasis. First, I^2^ is estimated imprecisely when few studies are available, and the values reported here should be regarded as indicative rather than as reliable quantifications of between-study inconsistency. Second, funnel plots based on two to four studies cannot meaningfully assess publication bias; their apparent symmetry in this review should not be taken as reassurance that small-study effects are absent.

## 5. Conclusions

PBM appears to be safe, feasible, and acceptable in patients with PD; however, the current body of evidence does not provide robust or conclusive support for clinically meaningful motor benefits. Meta-analysis results did not demonstrate a clinically meaningful treatment signal, though the limited number of eligible studies constrains the precision and generalizability of pooled estimates. These findings should be interpreted as insufficient evidence to establish a clinically meaningful motor benefit of PBM, rather than as evidence that no such benefit exists. With two to four trials contributing to each outcome and confidence intervals wide enough to remain compatible with effects on either side of the MCID, the present data cannot distinguish a genuinely null effect from a true effect that this evidence base is underpowered to detect. The interpretation of available findings is further constrained by substantial heterogeneity across studies in anatomical targeting, device type, wavelength, dosing parameters, study design, and outcome assessment, as well as by limited sample sizes and brief treatment durations. Future research will require adequately powered, rigorously designed trials with standardized intervention parameters, validated measures of target engagement, and clinically meaningful outcomes. Such studies will be essential to determine whether PBM has a reproducible and clinically relevant role in the motor management of PD.

## Figures and Tables

**Table 1:** General characteristics of included studies.

Study ID	Year	Country	Study Design	Sample Size	% of Women	% of Men	Age Years (Mean ± SD)	Hoehn & Yahr
Battesha et al. [40]	2025	Jordan	RCT	38	Intervention (26) Control (32)	Intervention (74) Control (68)	Intervention (57.11 ± 5.25) Control (57.37 ± 4.54)	I to IV
Bullock-Saxton et al. [41]	2021	Australia	RCT	20	Intervention (30) Sham (50)	Intervention (70) Sham (50)	Intervention (69.5 ± 7.3) Sham (65.9 ± 7.5)	Intervention (2.4 ± 0.9) Sham (2.2 ± 0.7)
Herkes et al. [39] and McGee et al. [38]	2023	Australia	RCT (Crossover design for the second paper)	40	Intervention (50) Sham (50)	Sham/Intervention (50) Intervention/Sham (50)	Intervention (73.3 ± 4.4) Sham (70.9 ± 5.8)	N/R
Hong et al. [45]	2021	Taiwan	Open-label single arm	18	Intervention (35.3)	Intervention (64.7)	Intervention (67.53 ± 8.83)	Intervention (2.35 ± 0.49)
Liebert et al. [23]	2021	Australia	Waitlist design	12	Intervention/Sham A (33) Intervention/Sham B (83)	Intervention/Sham A (66) Intervention/Sham B (16)	Intervention/Sham A (71.3 ± NR) Intervention/Sham B (63.8 ± NR)	Intervention/Sham A (2 ± 0.63) Intervention/Sham B (1.83 ± 0.4)
Saltmarche et al. [42]	2025	Canada	RCT (Double-blind, 3 stage)	63	Active (44) Sham (19)	Active (56) Sham (81)	NR (range 58–79)	2–3
Santos et al. (A) [43]	2019	Spain	RCT	35	Intervention (41) Sham (44)	Intervention (47) Sham (38)	Intervention (72 ± 7) Sham (70 ± 8)	Intervention (1.5 ± 0.6) Sham (1.5 ± 0.5)
Santos et al. (B) [44]	2025	Spain	RCT (4 arm, single blind)	53	Control (20) Exercise (9) PBM (23) Exercise + PBM (14)	Control (80) Exercise (91) PBM (77) Exercise + PBM (86)	Control (70 ± 7.2) Exercise (71.4 ± 8.2) PBM (72 ± 6.4) Exercise + PBM (68.2 ± 6)	1–2

Summary of the general characteristics of the included studies. NR = not reported.

**Table 2:** PBM intervention and dosimetric parameters.

Study	Wavelength (nm)	Device Architecture	Delivery Mode	Anatomical Target	Irradiance (mW/cm^2^)	Fluence (J/cm^2^)	Total Energy per Session (J)	Session Duration (min)	No. of Sessions
Battesha 2025 [40]	810	Transcranial (NR)	Continuous wave	Frontal, parietal	NR	NR	NR	NR	NR
Bullock-Saxton 2021 [41]	904	Handheld laser probe	Pulsed	Temporal, intraoral, central sagittal suture	306	10.2	42	NR	24
Herkes 2023 [43] and McGee 2023 [38]	810–904	Helmet, multifocal LED array (>20 sites)	Pulsed	Temporal, frontal, occipital ± intraoral	NR	NR	~1137.6	24	72
Hong 2021 [45]	NR	NR	NR	NR	6	~10.8	NR	NR	10
Liebert 2021 [23]	810–904	Helmet, multifocal LED array (>20 sites)	Pulsed	Temporal, frontal, occipital ± intraoral	NR	NR	NR	NR	36
Saltmarche 2025 [42]	810–904	Helmet, multifocal LED array (>20 sites)	Pulsed	Temporal, frontal, occipital ± intraoral	NR	NR	NR	NR	36
Santos 2019 [43]	670	Tabletop probe	NR	Bilateral temporal	60	21.6	NR	NR	18
Santos 2025 [44]	670	Tabletop probe	NR	Bilateral temporal	NR	NR	NR	NR	NR

Summary of the PBM intervention and dosimetric parameters. NR = not reported.

**Table 3:** Intervention protocols.

Study ID	Treatment Duration (Weeks)	Duration of Session	Session Frequency (per Week)	Duration of Intervention (Minutes)	Total Number of Sessions	Number of Follow-Ups	Combined Intervention	Type of Combined Intervention	Location (Research Center, Clinic, etc.)	Operator
Battesha et al. [40]	12 weeks	~12 min	5	12 min	60	1	Yes	Physiotherapy	Research center	Researcher
Bullock-Saxton et al. [41]	12 weeks	~12 min	3	NR	24	3	No	NA	Research center	Researcher
Herkes et al. [39] and McGee et al. [38]	12 weeks	24 min	6	12 min	72	2	No	NA	Research center	Researcher
Hong et al. [45]	12 weeks	30 min	7	NR	10	3	Yes	Hydrogen water	Research center	Researcher
Liebert et al. [23]	12 weeks	~20 min	3	35 min	36	2	No	NA	Research center/Home-based	Researcher/Patient
Saltmarche et al. [42]	8 weeks (Stage 1 RCT); up to 48 weeks (Stage 3)	24 min	3	24 min	24 (Stage 1); 48 (Stages 1 + 2)	4	Yes	Exercise	Home-based	Patient (self-administered)
Santos et al. (A) [43]	9 weeks	6 min	NR	9 min	18	1	No	NA	Research center	Researcher
Santos et al. (B) [44]	24 weeks	~17 min	2	~17 min	48	1	No (PBM arm)	NA	Research center (multicenter)	Researcher

Summary of the intervention protocols. NR = not reported.

**Table 4:** Extracted motor outcome data from included studies, stratified by outcome measure.

Study ID	MDS-UPDRS-III Baseline (Mean ± SD)	MDS-UPDRS-III Last Follow-Up (Mean ± SD)	TUG Baseline (Mean ± SD)	TUG Last Follow-Up (Mean ± SD)	TUG Motor Baseline (Mean ± SD)	TUG Motor Last Follow-Up (Mean ± SD)
Battesha et al. [40]	UPDRS * (14-item subset, not MDS-UPDRS-III) Intervention (37.26 ± 6.89) Control (37.37 ± 6.09)	UPDRS * (14-item subset, not MDS-UPDRS-III) Intervention (26.63 ± 5.62) Control (31.84 ± 5.77)	NR	NR	NR	NR
Bullock-Saxton et al. [41]	NR	NR	NR	NR	Intervention (11.4 ± 4.6) Sham (12.1 ± 3.5)	Intervention (10.2 ± 3.4) Sham (11.8 ± 4.2)
Herkes et al. [39] and McGee et al. [38]	Intervention (21.35 ± 9.43) Sham (26.00 ± 13.81)	Intervention (16.45 ± 9.48) Sham (20.47 ± 12.83)	NR	NR	NR	NR
Hong et al. [45]	Intervention (25.31 ± 13.08)	Intervention (22.03 ± 13.03)	NR	NR	NR	NR
Liebert et al. [23]	Intervention A/B (23.33 ± 10.65)	NR	Intervention A/B (8.91 ± 3.21)	Intervention A/B (7.70 ± 2.64)	Intervention A/B (9.03 ± 3.08)	Intervention A/B (8.28 ± 2.78)
Saltmarche et al. [42]	Active/Sham (28.1 ± 11.2)	Active Stage 1 (n = 29): 24.4 ± 9.3 Sham Stage 1 (n = 30): 27.7 ± 12.8	Active/Sham (11.4 ± 3.2)	Active (n = 29): 12.3 ± 4.2 Sham (n = 30): 11.0 ± 4.0	NR	NR
Santos et al. (A) [43]	Intervention (11 ± 5) Sham (9 ± 5)	Intervention (10 ± 4) Sham (9 ± 4)	Intervention (11.0 ± 2.6) Sham (12.6 ± 5.1)	Intervention (10.6 ± 2.5) Sham (12.8 ± 5.4)	NR	NR
Santos et al. (B) [44]	Control (n = 15): 9 ± 8.5 Exercise (n = 11): 10.3 ± 5.3 PBM (n = 13): 8.8 ± 8.0 Exercise + PBM (n = 14): 8.3 ± 6.0	Control: 13.2 ± 10.2 Exercise: 11.3 ± 9.5 PBM: 9.8 ± 7.5 Exercise + PBM: 10.1 ± 6.5 Between: Exercise vs. Control *p* = 0.003 * (r = 0.41); PBM vs. Control *p* = 0.030	Control: 9.7 ± 3.3 Exercise: 6.8 ± 2.0 PBM: 9.4 ± 3.2 Exercise + PBM: 7.4 ± 2.8	Control: 10.2 ± 3.3 Exercise: 6.7 ± 1.6 PBM: 9.1 ± 3.4 Exercise + PBM: 7.3 ± 3.0 Between: H(3) = 0.260, *p* = 0.100 (NS)	NR	NR

Values are reported as mean (SD). MDS-UPDRS-III = Movement Disorder Society-Unified Parkinson’s Disease Rating Scale Part III; TUG = Timed Up and Go; NR = not reported. Crossover design; Stage 1 between-group MD = 3.9 points favoring PBM. Prospective single-arm (waitlist) design; no parallel control group. * Battesha et al. 2025 reported UPDRS total score, not MDS-UPDRS-III [40].

**Table 5:** GRADE certainty of evidence for meta-analyzed motor outcomes.

Outcome	k	Risk of Bias	Inconsistency	Indirectness	Imprecision	Publication Bias	Certainty
MDS-UPDRS-III	4	Serious	Not serious (I^2^ = 20.6%)	Not serious	Very serious	Undetected	Very low
TUG	3	Serious	Serious (I^2^ = 55.1%)	Not serious	Very serious	Undetected	Very low
TMWT	2	Serious	Not serious (I^2^ = 0%)	Not serious	Very serious	Not assessed	Very low

GRADE certainty of evidence for the meta-analyzed outcomes.

## Data Availability

All data supporting the findings of this systematic review are available within the article and its Supplementary Materials.

## Supplementary Materials

The following supporting information can be downloaded at: https://www.mdpi.com/article/10.3390/brainsci16090925/s1, Supplementary Table S1, Supplementary Table S2, Supplementary Table S3, Supplementary Table S4, Supplementary Table S5, Supplementary Table S6; Supplementary Figure S1, Supplementary Figure S2, Supplementary Figure S3.

## Abbreviations

The following abbreviations are used in this manuscript:

PD	Parkinson’s disease
PBM	Photobiomodulation
LLLT	Low-level light therapy
RCT	Randomized controlled trial
PRISMA	Preferred Reporting Items for Systematic Reviews and Meta-Analyses
PROSPERO	International Prospective Register of Systematic Reviews
MeSH	Medical Subject Headings
RoB 2.0	Cochrane Risk-of-Bias tool, version 2
ROBINS-I	Risk Of Bias In Non-randomized Studies-of Interventions
MD	Mean difference
REML	Restricted maximum likelihood
HKSJ	Hartung–Knapp–Sidik–Jonkman
CI	Confidence interval
MDS-UPDRS-III	Movement Disorder Society-Unified Parkinson’s Disease Rating Scale, Part III
TUG	Timed Up and Go test
TMWT	Ten-Meter Walk Test
MCID	Minimal clinically important difference
DBS	Deep brain stimulation
SD	Standard deviation
