# Supplementary material for: Efficacy and Safety of Photobiomodulation Therapy for Motor Symptoms in Parkinson’s Disease: A Systematic Review and Meta-Analysis
Source: Brain Sci. 2026 Aug 31;16(9):925. doi: 10.3390/brainsci16090925 (PMC13604397; doi:10.3390/brainsci16090925)
Supplement: Supplementary file 1 [file brainsci-16-00925-s001.zip › brainsci-4478925-supplementary.pdf]

Supplementary Figures

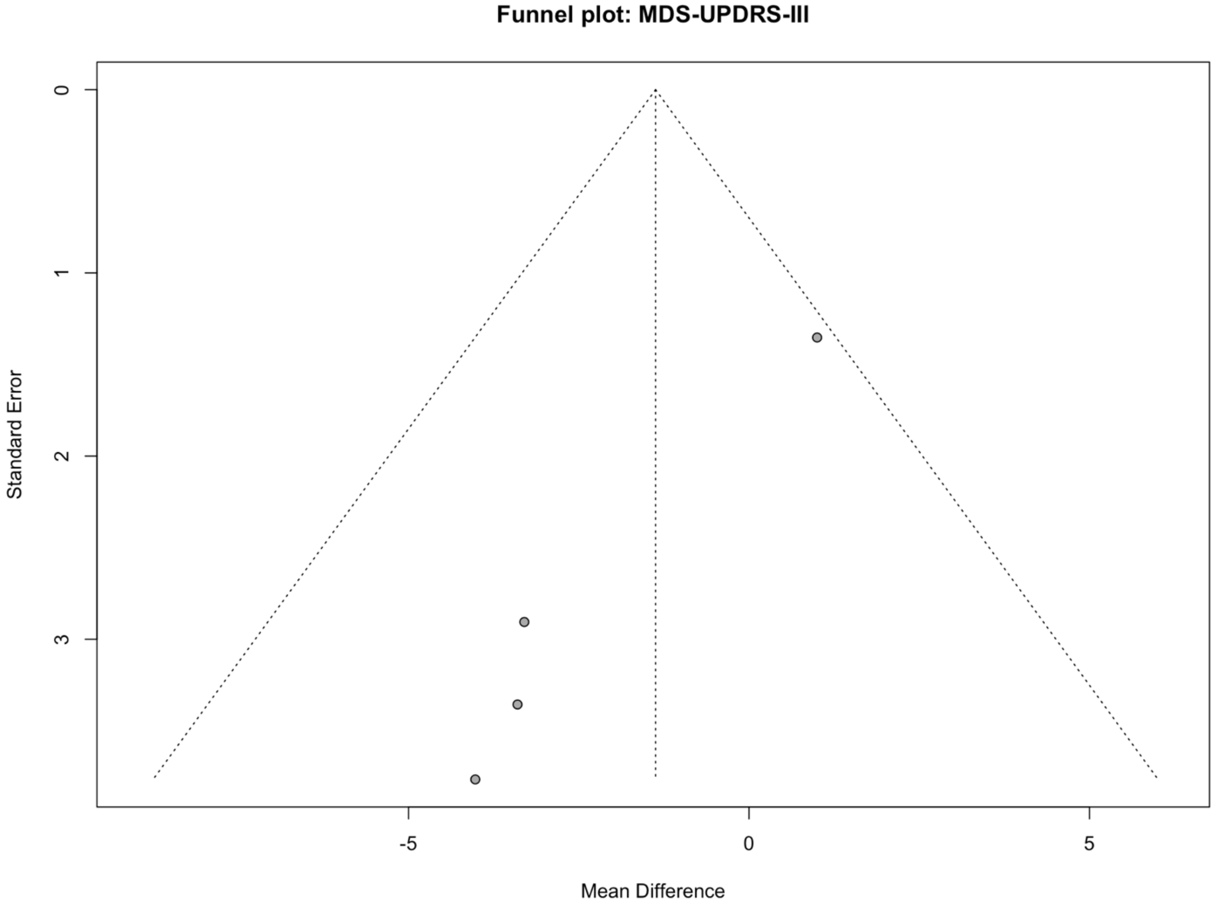

Supplementary Figure *S1. Funnel plot of MDS-UPDRS-III (k = 4).*

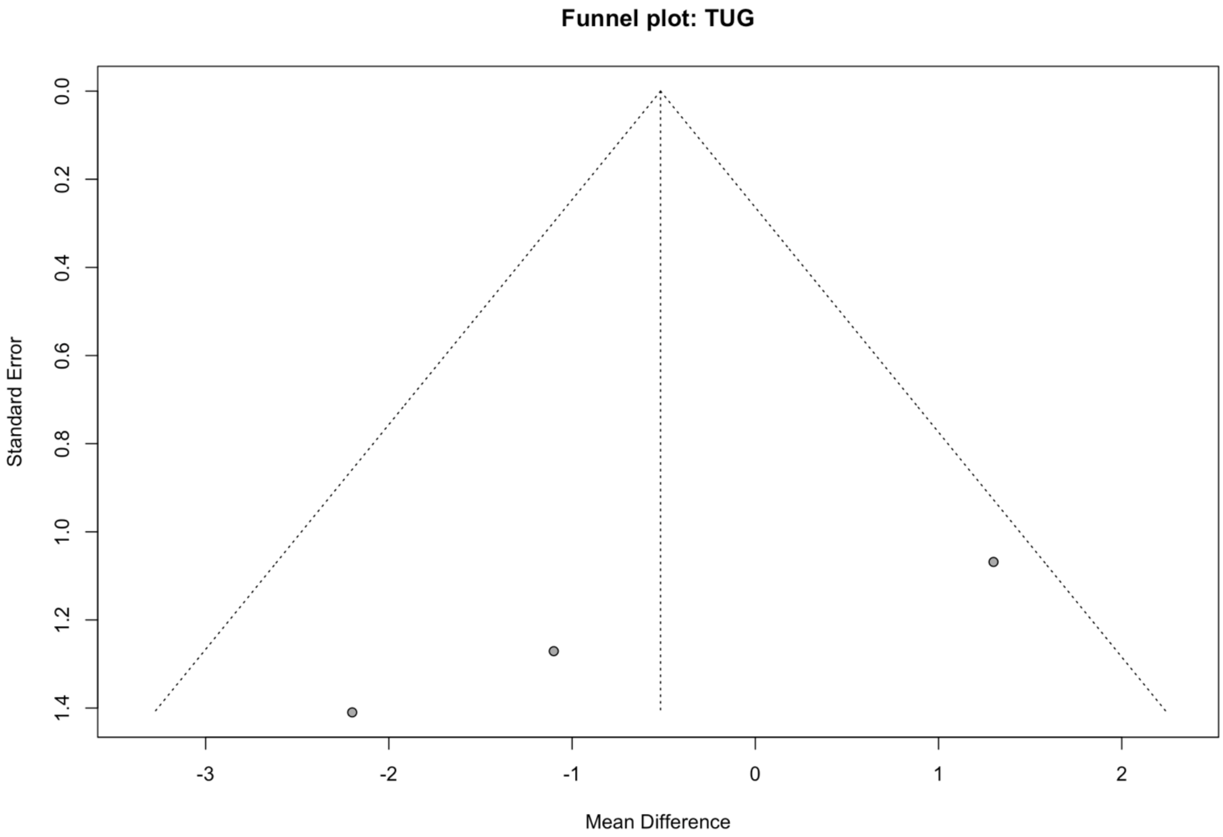

Supplementary Figure S2. Funnel plot of TUG (k = 3).

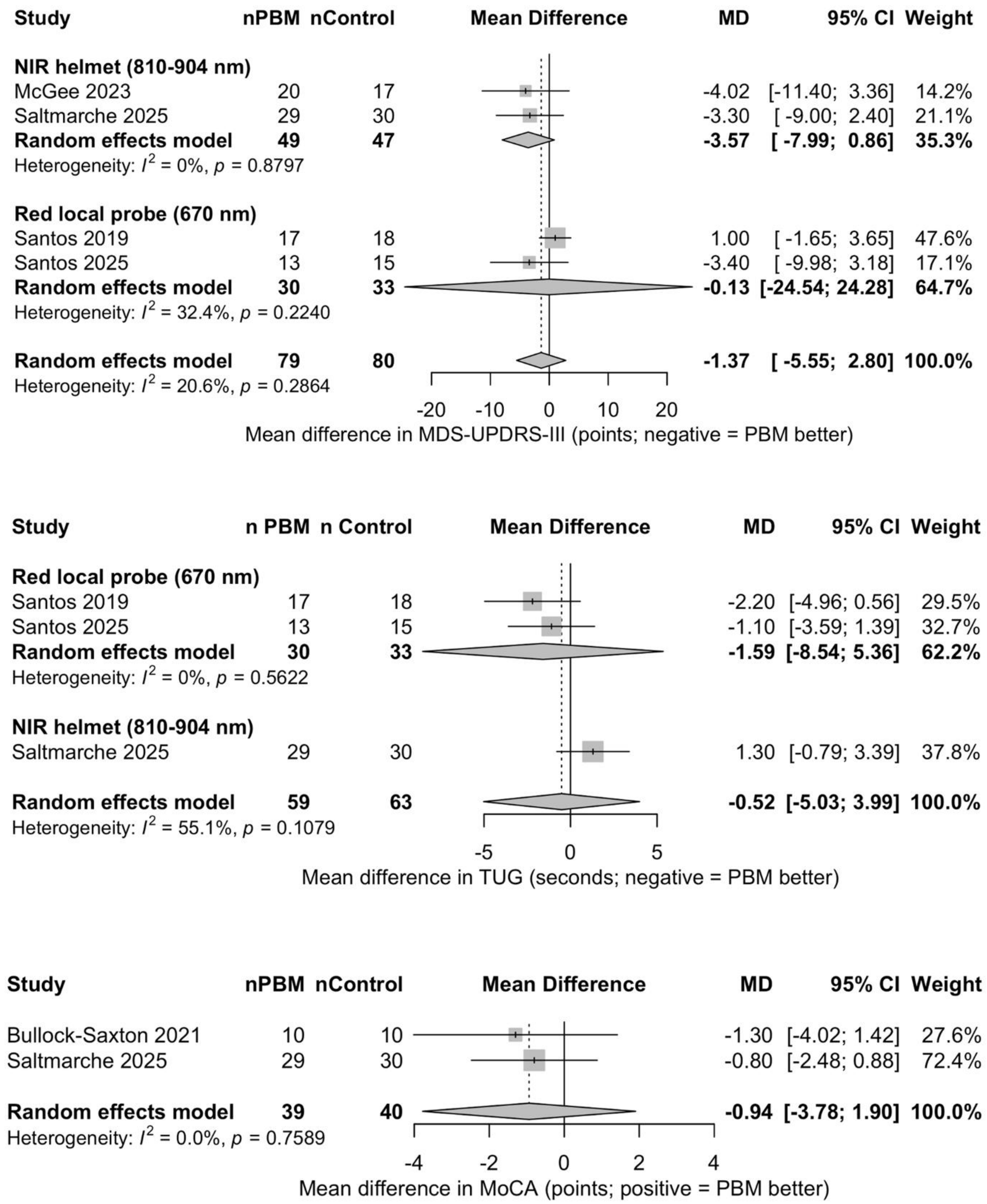

Supplementary Figure S3. Exploratory subgroup analysis by device/wavelength class. (A) MDS-UPDRS-III; (B) TUG; (C) MoCA, shown without stratification as both trials used the same device class. Negative values favour PBM for MDS-UPDRS-III and TUG; positive values favour PBM for MoCA.

Supplementary Table S1

**PubMed**	(“parkinson disease”[MeSH Terms] OR (“parkinson”[All Fields] AND “disease”[All Fields]) OR “parkinson disease”[All Fields]) AND (“low-level light therapy”[MeSH Terms] OR (“low-level”[All Fields] AND “light”[All Fields] AND “therapy”[All Fields]) OR “low-level light therapy”[All Fields] OR “photobiomodulation”[All Fields])
**Web of Science**	TS=(“parkinson disease” OR (“parkinson” AND “disease”)) AND TS=(“low-level light therapy” OR (“low-level” AND “light” AND “therapy”) OR “photobiomodulation”)
**Cochrane**	(“parkinson disease”:ti,ab,kw OR “parkinson’s disease”:ti,ab,kw OR (parkinson:ti,ab,kw AND disease:ti,ab,kw)) AND (“low-level light therapy”:ti,ab,kw OR photobiomodulation:ti,ab,kw OR “light therapy”:ti,ab,kw OR “PBM”:ti,ab,kw OR “LLLT”:ti,ab,kw)
**Embase**	(‘parkinson disease’/exp OR ‘parkinson disease’ OR (parkinson AND disease))<br>AND<br>(‘low level light therapy’/exp OR ‘low level light therapy’ OR (low-level AND light AND therapy)
**Google Scholar**	(Parkinson Disease OR Parkinson’s Disease OR PD) AND (Photobiomodulation OR Low-light Therapy) AND (Trial)

Supplementary Table S1. Complete search strategy for each database

Supplementary Table S2. PRISMA 2020 Checklist

Section & Topic	Item #	Checklist Item	Location Where Item Is Reported
**TITLE**			
**Title**	**1**	Identify the report as a systematic review.	Title page — “A Systematic Review and Meta-Analysis”
**ABSTRACT**			
**Abstract**	**2**	See the PRISMA 2020 for Abstracts checklist.	Structured abstract (Background/Objectives/Methods/Results/Conclusions),

			p.1. Recommend a separate pass against the PRISMA-A (Abstract) checklist before submission.
**INTRODUCTION**			
**Rationale**	**3**	Describe the rationale for the review in the context of existing knowledge.	Introduction, para. 4 ("Despite promising preclinical evidence and emerging clinical data, a significant gap remains…")
**Objectives**	**4**	Provide an explicit statement of the objective(s) or question(s) the review addresses.	Introduction, final paragraph
**METHODS**			
**Eligibility criteria**	**5**	Specify the inclusion and exclusion criteria for the review and how studies were grouped for the syntheses.	2.2 (inclusion/exclusion)
**Information sources**	**6**	Specify all databases, registers, websites, organisations, reference lists and other sources searched or consulted to identify studies. Specify the date when each source was last searched or consulted.	2.3 (5 databases; search cutoff May 7, 2026)
**Search strategy**	**7**	Present the full search strategies for all databases, registers and websites, including any filters and limits used.	2.3 + Supplementary Table 1
**Selection process**	**8**	Specify the methods used to decide whether a study met the inclusion criteria of the review,	2.4, para. 1 (Rayyan; two independent reviewers JVR/EPM)

		including how many reviewers screened each record and each report retrieved, whether they worked independently, and if applicable, details of automation tools used in the process.	
**Data collection process**	**9**	Specify the methods used to collect data from reports, including how many reviewers collected data from each report, whether they worked independently, any processes for obtaining or confirming data from study investigators, and if applicable, details of automation tools used in the process.	2.4, para. 2–3
**Data items**	**10a**	List and define all outcomes for which data were sought. Specify whether all results that were compatible with each outcome domain in each study were sought, and if not, the methods used to decide which results to collect.	**Introduction (final para.) + 2.4 — outcomes named (MDS-UPDRS-III, TUG, TMWT, safety) but not formally listed/defined as a discrete outcome set with timepoints.**
	**10b**	List and define all other variables for which data were sought. Describe	2.4, para. 2 (full variable list).

		any assumptions made about any missing or unclear information.	
**Study risk of bias assessment**	**11**	Specify the methods used to assess risk of bias in the included studies, including details of the tool(s) used, how many reviewers assessed each study and whether they worked independently, and if applicable, details of automation tools used in the process.	2.5
**Effect measures**	**12**	Specify for each outcome the effect measure(s) used in the synthesis or presentation of results.	2.6, para. 2 (mean difference)
**Synthesis methods**	**13a**	Describe the processes used to decide which studies were eligible for each synthesis.	2.6 — Studies were eligible for a given meta-analysis if they reported extractable post-intervention or between-group summary statistics for the same outcome measured.
	**13b**	Describe any methods required to prepare the data for presentation or synthesis, such as handling of missing summary statistics, or data conversions.	2.4 (Web Plot Digitizer; author contact)
	**13c**	Describe any methods used to tabulate or visually display results of individual studies and syntheses.	Implicit — realized in Tables 1–4 and Figures 4A–D (Results); not pre-specified as a method in 2.6.

	**13d**	Describe any methods used to synthesize results and provide a rationale for the choice(s). If meta-analysis was performed, describe the model(s), method(s) to identify heterogeneity, and software.	2.6, para. 2 (REML, HKSJ, I^2, R package meta v7.0-0)
	**13e**	Describe any methods used to explore possible causes of heterogeneity among study results (e.g. subgroup analysis, meta-regression).	2.6 – exploratory subgroup analysis by device/wavelength class; meta-regression not performed (k = 2–4)
	**13f**	Describe any sensitivity analyses conducted to assess robustness of the synthesized results.	2.6, para. 4 (exclusion of McGee 2023 for scale heterogeneity)
**Reporting bias assessment**	**14**	Describe any methods used to assess risk of bias due to missing results in a synthesis.	2.6, para. 4 (funnel plots). No formal asymmetry test (e.g., Egger's) is specified as a method.
**Certainty assessment**	**15**	Describe any methods used to assess certainty (or confidence) in the body of evidence for an outcome.	4.7 (new) — explicitly states GRADE (or an equivalent framework) was applied; certainty of pooled estimates is stated as low given study count and risk-of-bias profile.
**RESULTS**			
**Study selection**	**16a**	Describe the results of the search and selection process, ideally using a flow diagram.	3.1 + Figure 1 (PRISMA flow diagram)

	**16b**	Cite studies that might appear to meet the inclusion criteria, but which were excluded, and explain why.	The full-text screening/Rayyan export.
**Study characteristics**	**17**	Cite each included study and present its characteristics.	3.2 + Table 1
**Risk of bias in studies**	**18**	Present assessments of risk of bias for each included study.	3.3 + Figures 2–3
**Results of individual studies**	**19**	For all outcomes, present, for each study: (a) summary statistics for each group and (b) an effect estimate and its precision.	3.4 (narrative, per-study MD/CI/Cohen's d)
**Results of syntheses**	**20a**	For each synthesis, briefly summarise the characteristics and risk of bias among contributing studies.	3.5 lists contributing studies per synthesis; risk of bias is not explicitly cross-referenced within this section (see 3.3 separately).
	**20b**	Present results of all statistical syntheses conducted, with summary estimate, precision, heterogeneity, and direction of effect.	3.5, paras. 2–4 + Figures 4A–D
	**20c**	Present results of all investigations of possible causes of heterogeneity among study results.	3.5 and Supplementary Table 5 — exploratory subgroup analysis by device/wavelength class. Meta-regression not performed (k = 2–4).
	**20d**	Present results of all sensitivity analyses conducted.	3.5 and Supplementary Table 5 — subgroup results by device/wavelength class
**Reporting biases**	**21**	Present assessments of risk	3.5, para. 5 — funnel plots referenced (Supplementary Figs. S1–S2); manuscript explicitly states formal

		of bias due to missing results for each synthesis assessed.	asymmetry testing was not performed given the small number of studies.
**Certainty of evidence**	**22**	Present assessments of certainty in the body of evidence for each outcome assessed.	4.7 (new) — GRADE added for ceitainty of evidence.
**DISCUSSION**			
**Discussion**	**23a**	Provide a general interpretation of the results in the context of other evidence.	4.1–4.2
	**23b**	Discuss any limitations of the evidence included in the review.	4.6
	**23c**	Discuss any limitations of the review processes used.	4.6 focuses on evidence limitations (sample size, heterogeneity). Process-level limitations (English-language restriction, no grey-literature search, no formal **reporting-bias test) are not explicitly discussed.**
	**23d**	Discuss implications of the results for practice, policy, and future research.	4.5–4.6 + 5 (Conclusion)
**OTHER INFORMATION**			
**Registration and protocol**	**24a**	Provide registration information for the review, including register name and registration number, or state that the review was not registered.	2.1 (PROSPERO CRD420261411269)
	**24b**	Indicate where the review protocol can be accessed, or state that a protocol was not prepared.	2.1 (new) — Prospero registration number: CRD42026141126
	**24c**	Describe and explain any amendments to	2.1 (new) — describes the post-registration addition of the exploratory TMWT meta-analysis as the only amendment.

		information provided at registration or in the protocol.	
**Support**	**25**	Describe sources of financial or non-financial support for the review, and the role of the funders or sponsors.	Back matter — Funding statement ("This research received no external funding")
**Competing interests**	**26**	Declare any competing interests of review authors.	Back matter — Conflicts of Interest statement
**Availability of data, code and other materials**	**27**	Report which of the following are publicly available and where: data collection forms; extracted data; analytic data; analytic code; other materials.	Back matter — Data Availability Statement covers extracted/analysis data. R analysis script and data extraction forms are not separately mentioned as available.

Supplementary Table S3. Detailed MCIDs of each outcome.

Outcome	Pooled MD	MCID	MD/MCID ratio	95% CI relative to MCID
MDS-UPDRS-III	−1.37 points	3.25 points	−0.42	−1.71 to 0.86
TUG	−0.52 seconds	3.5 seconds	−0.15	−1.44 to 1.14
TMWT	−0.65 seconds	0.05 m/s	Not calculable	Different measurement units

Supplementary Table S4. Detailed assessment of RoB 2.

Study and design	D1	D2	D3	D4	D5	Overall	Rationale
Bullock-Saxton et al. 2021	Some concerns	Low	Low	Low	Some concerns	Some concerns	Allocation was described, but concealment was insufficiently reported. Missing outcome data were limited and unlikely to depend on the outcome. Standardized validated measures and blinded assessors supported low risk in outcome measurement. The broad outcome battery and limited accessible prespecification led to some concerns regarding selective reporting.
Herkes et al. 2023	Low	Some concerns	High	Low	Low	High	Computer-generated randomization and balanced groups supported low risk for D1. The sham-to-active extension and technical unblinding created some concerns for deviations from the intended intervention. Incomplete outcome data without adjustment for imputation or sensitivity analysis, and with dropout reasons potentially related to outcomes, resulted in a high risk for D3. Standardized assessments with blinded assessors and registered primary outcomes supported low risk for D4 and D5.
McGee et al. 2023	Low	Low	Low	Low	Some concerns	Some concerns	Independent randomization, blinding, and comparable groups supported low risk in D1 and D2. Most randomized participants contributed outcome data. Remote video assessment used a standardized validated measure with blinded assessors. The post-hoc analysis of multiple motor subdomains without an accessible prespecified analysis plan resulted in some concerns for selective reporting.
Santos et al. 2019	Low	Low	Low	Low	Some concerns	Low	Randomization and blinding were adequately described. Missing outcome data were limited and

							unlikely to bias the result. Standardized outcome measures and blinded assessment supported low risk for D4. Limited protocol and analysis-plan information and reporting of several outcomes resulted in some concerns for D5, but no high-risk domain was identified.
Battesha et al. 2025.	Some concerns	Some concerns	Low	Some concerns	Low	Some concerns	Randomization was stated and the trial was registered, but sequence generation and allocation concealment were insufficiently described. Adherence and deviations from the intended intervention were not fully documented. Outcome data were reported for the randomized groups. Blinding and assessor procedures were insufficiently described. Trial registration and reported outcomes supported low risk for selective reporting.
Saltmarche et al. 2025.	Low	Low	Low	Low	Low	Low	Randomization, allocation, blinding, outcome completeness, and standardized assessments were adequately described. Trial registration and prespecified outcomes supported low risk of selective reporting.
Santos et al. 2025	Low	Some concerns	High	Some concerns	Low	High	Randomization was reported and baseline groups were comparable. The four-arm design and exercise co-intervention created some concerns regarding deviations from the intended intervention and interpretation of the PBM contrast. The reduction from randomized to analyzed participants and limited missing-data handling resulted in high risk for D3. Blinding and outcome-assessment procedures were incompletely described across groups. Registration supported low risk for D5.

Supplementary Table S5. Detailed assessment of ROBINS-I

Study and design	D1	D2	D3	D4	D5	D6	D7	Overall	Rationale
Hong et al. 2021	Critical	Moderate	Serious	Serious	Low	Serious	Moderate	Critical	The lack of randomization and a concurrent comparator created critical risk of confounding. The open-label single-arm design also introduced serious concerns regarding intervention classification, deviations from the intended intervention, and outcome measurement. All participants reportedly completed the intervention, supporting low risk for missing data. The use of multiple outcomes and limited prespecification resulted in moderate concerns regarding selective reporting.
Liebert et al. 2021	Serious	Moderate	Low	Serious	Moderate	Serious	Moderate	Serious	The non-randomized waitlist design was vulnerable to baseline confounding and participant-selection bias. The intervention was clearly classified, supporting low risk for D3. Home treatment, open-label delivery, and differences in treatment exposure created serious concerns regarding deviations from the intended intervention and outcome measurement. Incomplete follow-up

									and limited reporting of the analysis plan resulted in moderate concerns for missing data and selective reporting.

Supplementary Table S6.

Outcome	Device / wavelength class	k	nPBM / nControl	MD (95% CI)	I^2	Test for subgroup differences
MDS-UPDRS-III (points)	NIR helmet, 810–904 nm	2	49 / 47	−3.57 (−7.99; 0.86)	0%	χ^2 = 3.11, df = 1, p = 0.078
	Red local probe, 670 nm	2	30 / 33	−0.13 (−24.54; 24.28)	32.4%	
TUG (seconds)	NIR helmet, 810–904 nm	1	29 / 30	+1.30 (−0.79; 3.39)	NA	χ^2 = 5.81, df = 1, p = 0.016
	Red local probe, 670 nm	2	30 / 33	−1.59 (−8.54; 5.36)	0%	
TMWT (seconds)	Red local probe, 670 nm	2	30 / 33	−0.65 (−1.78; 0.49)	0%	Not estimable

Supplementary Table S6. Exploratory subgroup analysis by device/wavelength class. Device architecture and wavelength were fully confounded and could not be examined separately. Not estimable for TMWT, as both contributing trials used the same device class.
